# Dose tracking assessment for image-guided radiotherapy of the prostate bed and the impact on clinical workflow

**DOI:** 10.1186/s13014-017-0815-y

**Published:** 2017-04-28

**Authors:** Lucia Clara Orlandini, Marianna Coppola, Christian Fulcheri, Luna Cernusco, Pei Wang, Luca Cionini

**Affiliations:** 1 Centro Oncologico Fiorentino, Radiation Oncology Department, Via A. Ragionieri 101, 50023 Florence, Italy; 2 Department of Radiation Oncology, Sichuan Cancer Hospital, No.55, the 4th Section, Renmin South Road, Chengdu, China; 3 Azienda Ospedaliera di Perugia, Health Physics Department, Piazzale Menghini 1, 06129 Perugia, Italy

**Keywords:** Radiotherapy, Image-guided radiotherapy, Intensi-modulated radiation therapy, Prostate cancer

## Abstract

**Background:**

The cumulative dose was compared with the planned dose among fourteen patients undergoing image-guided, intensity-modulated radiotherapy of the prostate bed. Moreover, we investigated the feasibility of adding dose tracking to the routine workflow for radiotherapy.

**Methods:**

Daily cone beam computed tomography was conducted for image-guided radiotherapy, and weekly cumulative delivered doses were calculated for dose tracking. Deformable image registration was applied to map weekly dose distributions to the original treatment plan and to create a cumulative dose distribution. The dose–volume histogram (DVH) cut-off points for the rectum and bladder and the planning target volume (PTV), were used to compare the planned and cumulative delivered doses. The additional time required by the departmental staff to complete these duties was recorded.

**Results:**

The PTV coverage of the delivered treatment did not satisfy the expected goal for three patients (V98% >98%). In another three patients, the DVH cut-off point for the bladder was higher than the limits, while for the rectum, treatment was as expected in all cases (two patients failed both their bladder constraints and the PTV coverage). Overall, four patients did not satisfy one or more criteria at the end of their treatment.

**Conclusions:**

A well-defined strategy for dose tracking assessment is feasible, would have minimal impact on the workload of a radiotherapy department, and may offer objective information to support radiation oncologists in making decisions about adaptive procedures.

## Background

Intensity-modulated radiotherapy (IMRT) techniques are now extensively used for radical treatments in patients with localized and locally advanced prostate cancer and in post-prostatectomy settings [1–3]. The main dosimetric advantage of IMRT is that high conformal dose distribution can be obtained, particularly in the presence of a concavely shaped target. This enables the safer delivery of higher doses to the target and better sparing of organs at risk (OARs), namely the rectum and bladder. However, the modifications or displacements of target volumes and OARs, relative to the radiation beam frequently occur because of patient positioning and different filling and pressure effects from OARs; these can result in significant dosimetric changes because of the sharp dose gradients between the target and normal tissue [4–7]. Unfortunately, such anatomical modifications may cause under-dosing of target organs and/or over-dosing of OARs.

Image-guided radiotherapy (IGRT) is commonly used to reduce setup errors in patient positioning and in the inter-fraction organ motion [8, 9]. Typically, the correction parameter involves moving the treatment table to re-position the shifted target point to the isocenter of the treatment device. Such a target-point correction is a widespread strategy in IGRT, and it has proven to be superior for treatment of tumour sites with less pronounced deformations. However, this technique does not correct the anatomical deformations, such as volume changes that result from modifications or filling differences in OARs, or variations in the planning target volume (PTV) that occur during the course of treatment. Previous studies have argued regarding the use of adaptive radiotherapy and how it can be applied to manage inter-fraction motion [10, 11] and have proposed offline strategies based on geometric and dosimetric feedback [12, 13]. To date, the complexity of the dosimetric adaptive radiotherapy means that it is not a common part of routine clinical practice.

We investigated how inter-fractional variations in patient anatomy affected the difference between planned and delivered doses in patients undergoing adjuvant IGRT-IMRT for prostate cancer. A detailed analysis was performed among fourteen patients undergoing prostate radiotherapy. The additional time required by a multidisciplinary group (therapists, radiation oncologists and medical physicists) to complete these duties was recorded to assess whether this working schedule was practicable in daily practice. We calculated the delivered dose weekly and compared the planned dose with the cumulative delivered dose at the end of the treatment course. Dose–volume histogram (DVH) cut-off points for the rectum and bladder, as well as the DVH target coverage, were used for the comparison. The additional time burden was analysed for each health professional involved. We aimed to investigate, retrospectively, the effectiveness and feasibility of including dose tracking in the clinical radiotherapy workflow in order to manage an appropriately timed adaptive procedure.

## Methods

### Patients selection and treatment planning optimisation

Fourteen patients with prostate carcinoma undergoing an adjuvant prostate treatment and who received IGRT-IMRT routinely were included in this study. Patients were treated according to our center’s protocol, with prescribed dose of 70 Gy delivered in fractions of 2 Gy daily. The clinical target volume (CTV) included the prostatic lodge and was defined following the RTOG consensus guidelines for postoperative radiation therapy [14]. The PTV was obtained by adding a 5-mm margin to the CTV in every direction, except for the posterior direction where a 4-mm margin was used to limit the dose to the rectum. Computed Tomography (CT) images were acquired with patients in the supine position, with their feet fixed in a support, and a wedge placed under the knee to avoid pelvic rotation; a 3-mm slice width was used. Magnetic resonance (MR) images were acquired in the same position, immediately after the CT scan. Patients were instructed to maintain an empty rectum and a full bladder for the CT scans, MR scans, and treatment. 2D-CT and MR images were rigidly registered with a multi modalities image registration software (Mirada XD, Mirada Medical Ltd, Oxford, UK); T2-weighted turbo spin echo transverse MR sequence was used for the fusion. The contouring of the prostate, rectum, and bladder was defined on the fusion image set by an experienced radiation oncologist (RO).

All patients were treated with 6 MV photon beams at seven equally spaced gantry angles. A Siemens Artiste linear accelerator (Siemens Medical Solution, Erlangen, Germany) equipped with a Megavoltage Cone Beam Computed Tomography (MV-CBCT) was used. The treatment planning was performed with a Pinnacle^3TM^ Step-and-Shoot IMRT system (P^3^IMRT, Version 9.0, Philips Medical Division, Madison, WI), using direct machine parameter optimisation. CBCT performed daily with the 6 MV photon beam of the linac was included in the treatment plan as an arc field [15, 16], and the daily imaging dose of the 8MU CBCT protocol was integrated into the prescribed dose [17]. In Table 1 the daily CBCT mean dose contribution to PTV mean dose is reported.

**Table 1:** Imaging contribution to the prescribed dose

	IMRT	MV-CBCT
MU	548 ± 84	8
Dose (cGy)	193.68 ± 0.48	6.31 ± 0.45

MUs and corresponding doses of the IMRT and MV-CBCT are reported

Plan optimisation was based on dose volume objectives for PTVs and on OAR constraints commonly used in clinical practice [18–20]; in Table 2 the adopted values are shown. We define Dx% as the dose (in Gy) received by x% of the volume, and Vy as the volume (in percentage) that receives y Gy. For the PTV, we defined D_max_ as the dose received by 1 cm^3^ of the target volume. We aimed to achieve a final plan delivering the prescribed dose to at least 98% of the tumour volume, but no more than 107% of the prescribed dose to no more than 1% of the tumour volume, while ensuring that OAR doses remained as low as achievable.

**Table 2:** Organs at risk dose-volume constraints and goal for the planning target volume (PTV) used for adjuvant prostate IMRT planning

Organ	Dose (Gy)	Volume (%)
Bladder	50	65
	65	50
	70	35
Rectum	40	60
	50	50
	60	35
	70	20
Femoral Heads	30	50
	50	10
Small Bowell	15	120 cc
	45	195 cc
PTV	Dpre ^a^	98

^a^ Prescription Dose

All constraints are based on a schedule of 2 Gy/fraction

To evaluate dose tracking, we used the Raystation Demo Version 4.5 Treatment Planning System (RaySearch Laboratories AB Stockholm, Sweden) with the machine used for treatment. The treatment planning delivered to the patient and approved by the RO carried out with Pinnacle was re-calculated with Raystation TPS on the reference CT images. This treatment planning was taken as the baseline for the treatment. The DVH of the planned treatment, with the resulting OAR cut-off points and percentage target coverage, was used to compare the differences between the cumulated delivered and approved doses.

### IGRT and retrospective dose tracking

Patients underwent daily IGRT with a megavoltage CBCT (MV-CBCT) mounted on the treatment machine. The MV-CBCT used the linear accelerator as the radiation source and a 40 × 40 cm^2^ amorphous-silicon electronic portal imaging for online volumetric imaging. The technical specifications of the MVision system have been described elsewhere in the literature [21]. The protocol involved a 200° gantry arc rotation from 270° to 110° of a 6-MV beam around the isocenter with a source-to-axis distance of 100 cm and a source-to-image distance of 145 cm. We acquired 200 two-dimensional projection images and combined them to reconstruct a volumetric CT image dataset with a field of view diameter of 27.4 cm. A protocol of 8 monitor units was adopted for prostate imaging. The daily CBCT scans before treatment were matched with the original CT scans by the RO and were used clinically to align the relevant anatomic structures of the pelvis including rectum and bladder to the anatomy. The patient’s isocenter was moved to the corrected position, as appropriate, and the daily displacement was recorded.

For each patient, the cumulative delivered dose was calculated weekly. A total of 7 CBCTs were included in the recalculation (assuming a reference CBCT for the entire week). To avoid discrepancies that were not strictly connected to the anatomical changes of the patient or their position, a first comparison was performed between the planned CT treatment and that calculated on day one of CBCT (CBCT_0_). Only patients for whom the difference (cut-off points for OARs and target coverage) between the dose calculated on the planning CT and the dose on CBCT_0_ was less than 1% were included in the study.

Rigid registration was done to take into account the shift applied to the treatment isocenter during online correction. A deformable registration, based on biomechanical modelling and finite element analysis, was then performed [22–25] with Raystation platform also used for dose tracking. The obtained deformation map was used to map the target and the OARs of the weekly CBCT to the original planning CT; a new geometry was then achieved for the selected structures. The same experienced RO who performed the daily matching also assessed the correctness of the propagated regions of interest on each slice of the CBCT, and adjusted them if necessary. A second deformable registration was then calculated based on these verified contours, and a final deformation map was obtained; the contours defined in both the CT images drove the deformable registration. After computing dose in the CBCT datasets, they were deformed back to the planning scan and accumulated for DVH analysis. The process of dose recalculation and dose accumulation in Raystation, as well as the dose deformation algorithms, have been discussed in detail in literature [26, 27].

The most significant rectal and bladder cut-off points for tracking during treatment were identified by the RO for each patient, and a comparison was performed between the accumulated delivered doses and the planned treatment doses. For each plan, the most significant DVH cut-off, among those reported in Table 1, was the one most likely not to be met at the end of the treatment course. The tolerance was given by respect to these OAR limits. For comparison of the PTV coverage, we used the D_98%_ and D_max_ value: for the D_98%_ parameter, we accepted a difference of 1.7 Gy (2.5% difference) between the delivered and prescribed dose; while for the D_max_ value, we accepted a 3 Gy difference.

Planned and delivered doses were compared based on the relative percentage dose differences (%diff D) as follows: %diff D = 100 × (D_98%D_− D_98%P_) D_98%P_; where D_98%P_ and D_98%D,_ represent the dose received by 98% of the volume from the planned and delivered treatment, respectively. A positive difference indicated that the delivered dose was higher than the planned dose, while a negative value indicated that the cumulated delivered dose was lower than the planned dose.

## Results

One of the fifteen patients screened in 7 months, was excluded from the analysis since an excessive variation of the rectum and bladder filling required repeated CBCT scans before almost each treatment session. The dose calculated on the CBCT0 gave more than 5% difference for rectum and bladder (V_40Gy_ and V_70Gy_, respectively) when compared with the one calculated on the planning CT.

**Fig. 1:**
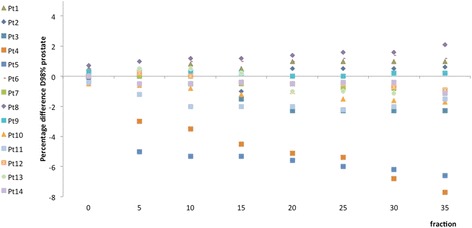
Prostate D_98%_ differences between the planned and the delivered dose during the treatment course. Prostate D_98%_ differences between the planned and the delivered dose during the treatment course. The differences were calculated as (D_98%P_-D_98%De_) x 100/D_98%P_. D_98%P_ and D_98%De_ represent the dose received by 98% of the volume from the planned and delivered treatment, respectively

The D_98%_ dose value for the PTV, resulting from the DVH of the delivered and planned treatment, is reported for each patient in column two of Table 3. Three of the fourteen patients (patients 3, 4 and 5) did not meet the criterion for PTV coverage, ending the treatment with D_98%_ lower than the prescribed dose of about 3.8, 5.7, and 4.2% respectively. The percentage dose difference for prostate coverage between the planned and delivered treatment is shown in Fig. 1. As can be observed, two of the three patients who ended their treatment with D_98%_ value out of the level of tolerance had already shown a trend towards under-dosing from the earliest fraction (fraction 5 for patients 4 and 5), whereas the under-dosing of patient 3 was harder to identify as the patient already started with a low value for D_98%_ (69 Gy) and ended with a percentage difference of 2.3% (67.4 Gy).

**Table 3:** The Dose received by 98% of prostate volume (D_98%_) at the end of the delivered treatment is reported in column 2, DVHs cut-off points V_70Gy_ for bladder in column 3, and V_40Gy_ and V_70Gy_ for rectum in columns 4 and 5 respectively; the first value shown is for the planned treatment and in brackets for the delivered one

Patient	PTV, D_98%_	Bladder, V_70Gy_	Rectum, V_40Gy_	Rectum, V_70Gy_
	D_98%_ (Gy)	Vol (%)	Vol (%)	Vol (%)
1	69.5 (70.2)	23% (25%)	30% (30%)	5% (4%)
2	71.5 (72.0)	38% (32%)	22% (22%)	2% (2.0%)
3^a^	69.0 (**67.4)**	26% (26%)	51% (49%)	16% (10%)
4	71.5 (**66.0)**	28% **(36%)**	26% (26%)	3% (3%)
5	71.8 **(67.0)**	15% (28%)	32% (35%)	0% (0.0%)
6	71.6 (72.5)	25% (3%)	27% (24%)	1% (1%)
7	70.6 (69.8)	30% (30%)	21% (20%)	3% (2%)
8	70.0 (71.5)	23% (29%)	26% (25%)	3% (%)
9	69.8 (70.0)	10% (12%)	13% (32%)	0% (0%)
10	70.7 (69.5)	25% (25%)	12% (11%)	1% (2%)
11	69.5 (68.5)	24% **(37%)**	20% (32%)	3% (4%)
12	70.2 (69.5)	20% (18%)	32% (32%)	0% (0%)
13	70.0 (69.2)	15% (10%)	55% (55%)	3% (1%)
14	70.5 (69.7)	23% (29%)	48% (40%)	7% (5%)

^a^ For Patient 3, V_50Gy_ =47% (79%) planned and delivered respectively

Values out of the expected values (2.5% of difference for D_98%_, out of the limits for OARs) are reported in bold

The D_max_ values obtained from the delivered treatments were comparable to those for the planned treatment: the difference between the maximum planned and delivered dose gave a median value of 2.15 ± 0.79 Gy maximum and minimum differences of 3.0 Gy and 0.5 Gy, respectively. These differences in the D_max_ absolute value were considered acceptable by the RO.

**Fig. 2:**
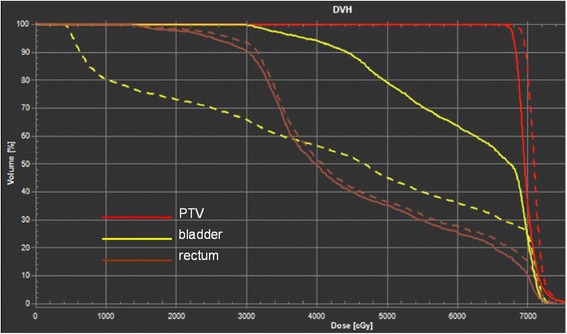
DVHs comparison of the delivered dose and planned dose for patient 3. DVHs comparison of the delivered dose (*solid line*) and planned dose (*dashed lines*) for patient 3. The curves for bladder, rectum and planning target volume (PTV) are reported with *yellow*, *brown* and *red lines* respectively

Concerning the OARs, the RO identified V_40Gy_ for the rectum and V_70Gy_ for the bladder as the most significant DVH cut-off points for tracking during treatment; this was chosen because their initial values were the closest to the respective limit and could potentially exceed it. V_70Gy_ for rectum is a strong predictor of rectal toxicity and was monitored despite the fact that the values obtained were far from exceeding the limit. The results of the clinical optimisation are reported in Table 3, columns 3, 4 and 5. The values obtained at the end of the accumulated delivered treatment are shown in brackets. Two of the fifteen patients failed to meet the bladder constraints (patients 4 and 11), while all patients were within the rectum constraints by the end of treatment. For patient 4, the V_70Gy_ value for the bladder increased from 28 to 36%, so this patient failed to meet two criteria (i.e., the target coverage and the bladder constraints). For patient 11, the V_70Gy_ for the bladder rose from 24 to 37%. Although patient 3 first met the cut-off parameter selected by the RO for the bladder (V_70Gy_), 79% of his bladder volume unfortunately received a dose of 50 Gy at the end of the treatment, so he failed both the target dose coverage and the bladder constraint; Fig. 2 shows the comparison of the DVHs for the planned and delivered treatment in this patient.

**Fig. 3:**
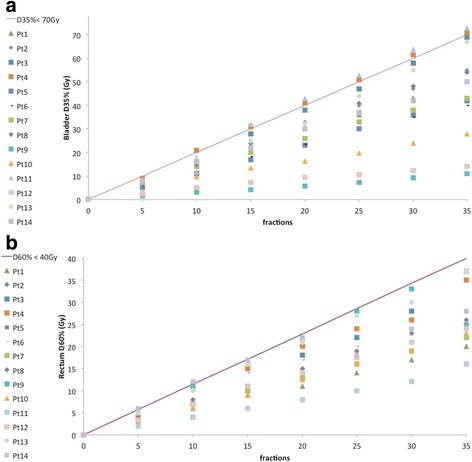
Evaluation of the cumulative dose delivered to the rectum and to the bladder. The cumulative dose received by 35% of the bladder volume (D_35%_) and the dose received by 60% of the rectum volume (D_60%_) is reported vs. the treatment fractions in **a** and **b** respectively. The *solid line* represents the cut off value for the rectum (D_60%_ < 40 Gy) and for the bladder (D_35%_ < 70 Gy)

The cumulative dose delivered to 35% of the bladder volume (D_35%_) and 60% of the rectum volume (D_60%_) vs the treatment fractions for every patient of the study, were reported in Fig. 3a, and b respectively; the solid lines indicate the cut-off values for the rectum and the bladder (D_60%_ <40 Gy and D_35%_ <70 Gy, respectively). Regarding the overall pass criteria (minimum required PTV coverage and within the OAR constraints), four of fourteen patients did not satisfy one or more criteria by the end of their radiotherapy.

The time needed for the dose tracking procedure averaged 2 h for the RO and 4 h for the medical physicist, with no extra time being needed by the therapist (they were not directly involved). In particular, it took about 15 min for the RO to complete each CBCT, including the time needed for assessment of the rigid registration, adjustment and assessment of the contours after mapping regions of interest, and final assessment of the deformable registration. By contrast, it required the medical physicist approximately 4 h to reconstruct and evaluate the cumulative dose. The commonly accepted IGRT procedure required a maximum time of 10 min for each CBCT–CT online matching assessment for the RO (if the therapist called him at the beginning of the CBCT procedure) and for the therapist, while no extra time to the workflow of the medical physicist.

## Discussion

The low image contrast of the MV CBCT can appear a limitation if compared with the kV-CBCT although the results of our study were not affected. Other authors dealing with MV-CBCT in clinical situations applied and successfully used a similar protocol [16, 21, 28]. Our ROs, based on their clinical experience, verified that the image quality of 8MU CBCT protocol adopted was adequate for soft tissue visualization nor influence the registration process with the kV planning CT.

The acceptance criterion of D_98%_ >97.5% for the PTV corresponds to an acceptable difference of 1.7 Gy in the delivered and planned dose. Considering that the prescription was 70 Gy we expected D_98%_ dose values >68.3 Gy. However, three patients did not meet their respective criteria (patients 3, 4, and 5). The PTV was used as the main target by our radiation oncologist throughout treatment, as indicated in Prescribing, Recording and Reporting Photon Beam Therapy (Report 62) by the International Commission on Radiation Units & Measurements (ICRU) [29], which states that coverage must be in a range between 95% and 107% of the prescribed dose.

For the treatment of prostate cancer, we tend to achieve better results than the ICRU recommendation and the D_98%_ parameter is used. However, variation in the organs surrounding the prostate bed may easily lead to the target parameters not being met. Neither patient 3 not patient 4 met the bladder constraint criterion, with patient 4 showing an increase in the V_70Gy_ value for the bladder from 28 to 36%, and patient 3 showing an increase in D_50Gy_ from 47 to 79%. Even though this was not the initial cut-off point selected to track the treatment, it cannot be ignored. As extensively reported in the literature, surrounding organs may present significant day-to-day variations in shape if they remain uncontrolled during routine treatment. As reported by Frank [4], most patients do not have a full bladder at the time of treatment because this can be difficult to maintain. Even if our patients followed strict rules for treatment preparation (500 mL of water 30 min before treatment and defecation to ensure a full bladder and an empty rectum, respectively), some may still experience difficulties. The use of rectal balloons can help in prostate immobilization and rectal toxicity reduction, and its use is highly investigated and discussed [30]. However, the technical difficulty of placing an endorectal balloon on a daily basis, as well as the patient discomfort associated with this procedure has been weighed against the benefit of the use of this device in our center.

As can be clearly seen in Fig. 3a, the acceptable bladder values (represented by the solid line) had been exceeded by fractions 10 and 15 in patients 4 and 11, respectively. For these two patients, the discrepancies were already present during verification, before treatment and were ascribed to poor compliance in maintaining bladder volume. Despite this discrepancy noted during CBCT0 imaging the patients were enrolled as the patient criteria selection was not affected. It is likely that patient 3 underwent the first CT planning with a full bladder, but was not able to reproduce it again. This different organ configuration may have led to over-dosing of the bladder and an under-dosing of the prostate because of changes in the location of the organs from treatment planning. These results can be extrapolated out to the last intended fraction to help the clinicians to assess if the original treatment goals would be achieved. The final treatment outcomes may have been improved with a re-planning procedure during treatment. Different authors [10, 11, 31, 32] analyzed the impact of an adaptive procedure in a clinical treatment, and demonstrated the effectiveness of offline dose compensation technique in IGRT prostate cancer. A single re-planning performed when the dose accumulation process exceeds the clinician thresholds may be necessary during the course of radiotherapy to remediate timely a deviation of the delivered treatment from the planned one. The time of the re-planning may be hard to identify, and therefore an optimised strategy of dose tracking must be set up. An early identification of OARs and cut-off points for acceptable upper dose limits, the set-up of the level of intervention (clinician thresholds for altering the planned treatment), and a reasonable frequency of the dose accumulation process, are the main issues discussed in our study. The additional time needed for dose tracking assessment was considered reasonable and less than that needed for an IGRT procedure, resulting in an acceptable impact on workflow in the radiotherapy department.

There are many important and practical concerns to be addressed for a successful dose tracking procedure, for example, intrafraction motion, organ contouring, the accuracy of the image registration and the reliability of deformable organ registration. Currently, many researchers are being carried out to manage these uncertainties, which are beyond the scope of this study. The results of our research must be understood within the insightful limitation of this study. The patients were enrolled only if no significant systematic error was found during the CBCT0 imaging; therefore, the results cannot be generalized to the entire population of men. The study involved a limited number of patients coming from a single institution, therefore it can be considered a small study and the contouring even if performed by the same RO following international guidelines was not validated by multi-institutional quality assurance program. However, the results show that deviations from the initial conditions can arise and that these deviations can lead to dosimetric differences over the target and OARs that cannot be neglected.

The intention of the authors is to go on with the study, investigating how the time of re-planning following the dose tracking procedure proposed can affect the comparison of normal tissue control probability and tumour control probability of the planned and delivered treatments.

## Conclusions

In this group of fourteen patients undergoing adjuvant radiotherapy for prostate cancer, comparison of the cumulative dose delivered to the patient and the approved planning dose showed that there was a deviation from the accepted initial conditions of the PTV coverage, the OAR constraints, or both in four patients (>28%). In these cases, a re-planning during the course of treatment may have avoided these discrepancies. We conclude supporting the utility and feasibility of dose tracking assessment in a radiotherapy routine as it offers objectives information to support radiation oncologists in making decisions about adaptive procedures with an acceptable added workload.

## Abbreviations

CBCT: Cone beam computed tomography
CT: Computed tomography
CTV: Clinical target volume
DVH: Dose volume histogram
IGRT: Image-guided radiotherapy
IMRT: Intensity-modulated radiotherapy
MR: Magnetic resonance
MV-CBCT: Megavoltage computed tomography
OARs: Organs at risk
PTV: Planning target volume
RO: Radiation oncologist
